# Plasma Levels of CGRP During a 2-h Infusion of VIP in Healthy Volunteers and Patients With Migraine: An Exploratory Study

**DOI:** 10.3389/fneur.2022.871176

**Published:** 2022-04-01

**Authors:** Lanfranco Pellesi, Mohammad Al-Mahdi Al-Karagholi, Roberto De Icco, Basit Ali Chaudhry, Cristina Lopez Lopez, Josefin Snellman, Jens Hannibal, Faisal Mohammad Amin, Messoud Ashina

**Affiliations:** ^1^Danish Headache Center, Department of Neurology, Rigshospitalet Glostrup, Faculty of Health and Medical Sciences, University of Copenhagen, Copenhagen, Denmark; ^2^Headache Science & Neurorehabilitation Center, Istituto Neurologico Nazionale a Carattere Scientifico (IRCCS) Mondino Foundation, Pavia, Italy; ^3^Department of Brain and Behavioral Sciences, University of Pavia, Pavia, Italy; ^4^Novartis Pharma A.G, Basel, Switzerland; ^5^Department of Clinical Biochemistry, Bispebjerg Frederiksberg Hospital, Faculty of Health Sciences, University of Copenhagen, Copenhagen, Denmark; ^6^Department of Neurorehabilitation and Traumatic Brain Injury, Rigshospitalet, Copenhagen, Denmark

**Keywords:** autonomic, headache, PACAP38, pain, parasympathetic system

## Abstract

**Introduction:**

The activation of perivascular fibers and the consequent release of vasoactive peptides, including the vasoactive intestinal polypeptide (VIP), play a role in migraine pathogenesis. A 2-h infusion of VIP provoked migraine, but the mechanisms remain unknown. We investigated whether 2-h infusion of VIP caused alterations in plasma levels of the calcitonin gene-related peptide (CGRP) and whether any changes might be related to the induced migraine attacks.

**Materials and Methods:**

We enrolled individuals with episodic migraine without aura and healthy participants to randomly receive a 2-h infusion of either VIP (8 pmol/kg/min) or placebo (sterile saline) in two randomized, placebo-controlled crossover trials. We collected clinical data and measured plasma levels of VIP and CGRP at fixed time points: at baseline (T_0_) and every 30 min until 180 min (T_180_) after the start of the infusion.

**Results:**

Blood samples were collected from patients with migraine (*n* = 19) and healthy individuals (*n* = 12). During VIP infusion, mixed effects analysis revealed a significant increase in plasma CGRP (*p* = 0.027) at T_30_ (vs. T_180_, adjusted *p-*value = 0.039) and T_60_ (vs. T_180_, adjusted *p*-value = 0.027) in patients with migraine. We found no increase in plasma CGRP during VIP-induced migraine attacks (*p* = 0.219). In healthy individuals, there was no increase in plasma CGRP during VIP (*p* = 0.205) or placebo (*p* = 0.428) days.

**Discussion:**

Plasma CGRP was elevated in patients with migraine during a prolonged infusion of VIP, but these alterations were not associated with VIP-induced migraine attacks. Given the exploratory design of our study, further investigations are needed to clarify the role of CGRP in VIP-induced migraine.

**Clinical Trial Registration:**

ClinicalTrials.gov, identifier: NCT03989817 and NCT04260035.

## Introduction

The vasoactive intestinal polypeptide (VIP) is a 28 amino acid neuromodulator and neurotransmitter belonging to the glucagon/secretin superfamily of peptides (1, 2). It is mainly released from the cranial parasympathetic fibers through the sphenopalatine ganglion and the otic ganglion (3). VIP is often co-released with other neuropeptides and could, therefore, exert some effects on other relevant neurotransmitter systems in the cranium (4). A double-blind, crossover study demonstrated that VIP induced migraine attacks in a large proportion of patients with migraine (5). At present, the mechanisms underlying VIP-induced migraine remain unknown.

Migraine is characterized by the activation of the trigeminovascular system, leading to the release of signaling peptides including the calcitonin gene-related peptide (CGRP) (6). CGRP is a potent vasoactive peptide released from unmyelinated perivascular fibers that may activate and sensitize perivascular nociceptors, potentially resulting in the sensation of head pain (7). It has been hypothesized that the release of VIP enhances extravasation of plasma proteins and the consequent release of CGRP from perivascular fibers (8, 9). Theoretically, the cranial parasympathetic outflow might activate perivascular nerve endings, inducing CGRP release and sensitizing the neurons in the spinal trigeminal nucleus (9). To date, the relationship between VIP and CGRP in humans and its potential relevance to migraine has never been investigated.

We previously described the headache-inducing abilities of a 2-h infusion of VIP in patients with migraine and healthy individuals (5, 10). In this study, we hypothesized that a 2-h infusion of VIP can elevate plasma levels of CGRP. In addition, we hypothesized that elevated plasma levels of CGRP are associated with experimentally induced migraine attacks.

## Materials and Methods

The participants were recruited between June 2019 and September 2020 from the Danish Headache Center (Rigshospitalet, Glostrup). We enrolled male and female patients, aged between 18 and 40 years old, with a diagnosis of migraine without aura as defined by the International Classification of Headache Disorders (ICHD-3) (11) with a frequency of 1–6 migraine days per month. Only women who used secure prevention (oral contraception or intrauterine device) were included in the studies. Healthy male and female individuals that were included were aged between 18 and 45 years old. Exclusion criteria for patients with migraine included any other type of headache (including more than 3 days of tension-type headache per month) as defined by the ICHD-3; anamnestic or clinical evidence of cardiovascular diseases, mental illnesses, smoking or substance abuse; daily intake of any medicine apart from oral contraceptives; and pregnant or breastfeeding women. Exclusion criteria for healthy participants were a history of migraine or any other type of headache (except tension-type headache no more than once a month in the last year); first-degree relatives diagnosed with migraine or any other type of headache (except tension-type headache); anamnestic and/or clinical evidence of cardiovascular diseases and mental illnesses; smoking or substance abuse; daily intake of any medicine other than oral contraceptives; and pregnant or breastfeeding women. The present studies (H-19075630 and H-18050862) were approved from the Regional Health Research Ethics Committee of the Capital Region and conducted in accordance with the Helsinki Declaration of 1964 with later revisions. All participants were informed that VIP might induce headache, but the headache characteristics or timing were not discussed.

### Experimental Design

Two randomized, double-blind, placebo-controlled, crossover trials were conducted. All participants were randomly allocated to receive a 2-h infusion of VIP (8 pmol/kg/min) or placebo (sterile saline). Infusions were performed using a volume-controlled infusion pump (Braun Perfusor, Melsungen, Germany) on two different study days, separated by at least 7 days (healthy individuals) or 14 days (patients with migraine). Before the experiments, a full medical examination was conducted for all enrolled individuals. They arrived headache free and non-fasting at the center. The intake of coffee, tea, cocoa, tobacco, alcohol, or other methylxanthine-containing beverages or foods was not allowed for 8 h before the start of the infusion. All procedures were performed in a quiet room at 25°C. The participants were placed in the supine position, and two venous catheters (Venflon, Braun Melsungen, Germany) were inserted into the antecubital vein of the left and right forearm for drug infusion and blood sampling. Headache intensity, accompanying symptoms, vital signs, and any side effect were recorded at T_−10_, at baseline (T_0_), and every 10 min after the start of the infusion until 200 min (T_200_). After finishing the experimental procedures, the participants were discharged and asked to complete a headache diary until 12 h after the start of the experiment. On both study days, a pregnancy test was performed in women of childbearing age. The experiments were postponed if the participant had experienced any kind of headache or used analgesics 48 h before the start of the experiment.

### Blood Collection and Processing

Before the sampling, 5 ml of blood were collected through a venous catheter and discarded. Blood was sampled through a 20 ml syringe at fixed time points: at T_0_ and every 30 min up to T_180_. The venous catheter was flushed with saline after sampling. The blood was then moved into pre-cooled lithium heparin tubes containing aprotinin (Trasylol®) for VIP evaluation and standard ethylenediaminetetraacetic acid tubes for CGRP evaluation. All tubes were gently inverted several times and stored at 5°C for 20 min until centrifugation (1,600 g for 10 min). Then, plasma was moved to cryotubes (Thermo Fisher Scientific, Jiangsu, China) and stored at −80°C for further analysis. At the moment of the analysis, laboratory technicians were blinded to the randomization lists.

### Plasma VIP

In healthy volunteers, VIP was measured by a previously described radioimmunoassay (12). In patients with migraine, VIP was measured by a different radioimmunoassay (Diasource, Louvain-la-Neuve, Belgium). The commercial assay differs from the original assay primarily because the detection of plasma VIP is performed without a previous plasma extraction. The extraction procedure is a requirement for the original assay, but often reduces the amount of measured peptide (12).

### Plasma CGRP

Concentrations of plasma CGRP were determined by radioimmunoassay using antibody AB 4-2905 and α-CGRP as calibrator (13), as previously described (14, 15).

### Statistical Analysis

All data are presented as mean and range, mean ± standard deviation, median and interquartiles (IQR), or numerosity and percentage (%). The primary exploratory endpoints were (a) the changes of plasma CGRP and VIP during VIP and placebo days in patients with migraine and healthy individuals and (b) the changes of plasma CGRP during experimentally induced migraine attacks. Regarding (a), we used two separate and independent mixed effects analysis to compare the plasma concentration of VIP and CGRP among each time point during VIP and placebo days in patients with migraine and healthy individuals. Lack of sphericity was adjusted with the Greenhouse-Geisser correction. Multiple comparisons were corrected using Tukey's *post-hoc* method and adjusted *p*-values were reported. Regarding (b), we calculated the sum of differences between plasma CGRP at the baseline and the following time points after VIP and placebo infusions. In this way, we obtained a summary score of the changes of plasma CGRP during VIP and placebo days. Then, we compared the scores between migraine days vs. no migraine days by the Wilcoxon matched pairs signed rank test. The secondary exploratory endpoint was the baseline difference of plasma CGRP between patients with migraine and healthy individuals. Plasma concentrations of CGRP at the baseline were compared by the Kruskal–Wallis test. When two baseline values were obtained from the same patient, the mean of the two values was considered for the comparison. We used R version 4.0.2 (R Project for Statistical Computing) and Prism 9.0.2 (GraphPad) for statistical analysis and graphs. All *p*-values were two-sided and considered significant if <0.05.

## Results

In total, 21 patients with migraine and 12 healthy individuals completed the study (5, 10). The frequency of baseline migraine days ranged from 1 to 6 per month. During the observational period (0–12 h), fifteen out of 21 patients with migraine (71%) developed migraine-like attacks after VIP, compared with one patient (5%) after taking placebo. Regarding healthy individuals, 8 out of 12 (67%) reported headache after VIP, compared to 1 out of 12 (8%) after placebo during the observational period (0–200 min). After VIP, three healthy individuals (25%) reported a migraine-like attack. There was no carryover or period effect for any variable in both studies. Due to some random effects, including the inability to collect blood samples in all participants and technical issues in the laboratory, data were missing in 21% (54/254) of the samples collected in patients with migraine and in 35% (59/168) of the samples collected in healthy volunteers. Considering the primary exploratory endpoint, we analyzed blood samples from 19 patients with migraine and 12 healthy volunteers. Regarding the second primary exploratory endpoint, we only considered patients migraine without missing values who reported a migraine attack in the hospitalization period during the VIP day, but not during the placebo day (*n* = 6). The study designs are displayed in Supplementary Figures 1, 2.

### CGRP and VIP in Patients With Migraine

**Figure 1:**
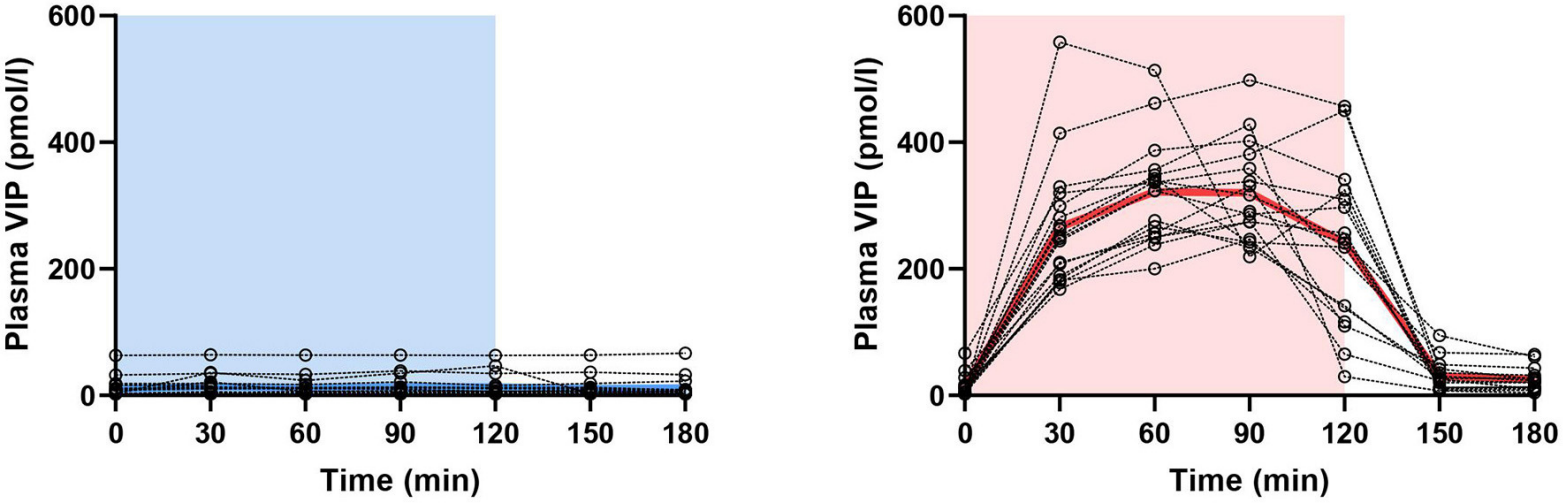
Changes in plasma concentration of vasoactive intestinal polypeptide (VIP) during and after 2-h infusion of placebo (*p* = 0.240) or VIP (*p* < 0.001) in patients with migraine. The light blue area represents placebo infusion, while the red area represents VIP infusion. Dotted lines represent individual values, while thick lines show mean concentrations.

**Figure 2:**
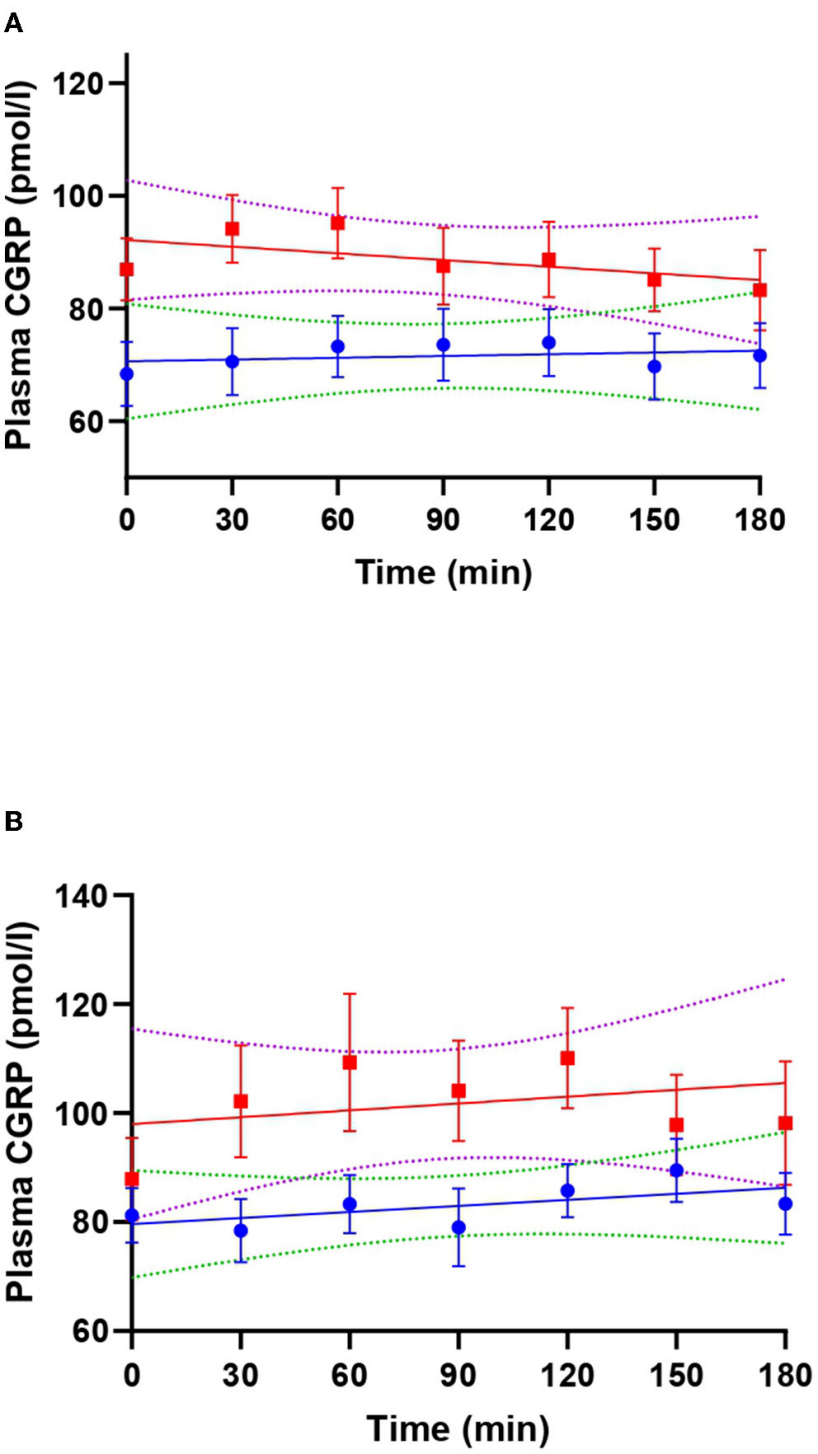
Linear regression lines of plasma calcitonin gene-related peptide (CGRP) during VIP (red) and placebo days (blue) in patients with migraine **(A)** and healthy individuals **(B)**. Dotted lines represent the 99% confidence bands of the best fit-line. In **(A)**, T_30_ (adjusted *p*-value = 0.039) and T_60_ (adjusted *p*-value = 0.027) were significantly different from T_180_ during VIP days.

On VIP days, plasma VIP was significantly higher compared to the baseline (*p* < 0.001), but not on placebo days (*p* = 0.240) (Figure 1). Peak concentration of plasma VIP was 321.7 ± 81.0 pmol/l, measured at T_60_ during the VIP infusion. Results from the mixed effects analysis found a significant increase in plasma CGRP during VIP infusions (*p* = 0.027), but not during placebo (*p* = 0.657). Tukey's *post-hoc* revealed that plasma CGRP was increased at T_30_ (vs. T_180_, adjusted *p*-value = 0.039) and T_60_ (vs. T_180_, adjusted *p*-value = 0.027) during VIP infusion. We found no differences regarding plasma levels of CGRP during VIP-induced migraine attacks that were observed in the hospitalization period (*n* = 6, *p* = 0.219) compared with placebo days in the same individuals. The mean plasma concentrations of CGRP in patients with migraine are reported in Table 1. A simple linear regression of plasma levels of CGRP during VIP and placebo days is displayed in Figure 2A.

**Table 1:** Plasma CGRP (pmol/l) and absolute number (*n*) of participants who have completed the analysis.

	**Patients with migraine**		**Healthy individuals**	
	**Placebo**	**VIP**	**Placebo**	**VIP**
0	68.4 ± 24.3 (18)	86.9 ± 24.1 (19)	81.2 ± 15.1 (9)	87.9 ± 21.3 (8)
30	70.6 ± 25.9 (19)	94.2 ± 25.4 (18)	78.4 ± 17.4 (9)	102.1 ± 27.2 (7)
60	73.3 ± 23.0 (18)	95.1 ± 25.2 (16)	83.3 ± 16.1 (9)	109.3 ± 33.3 (7)
90	73.6 ± 27.2 (18)	87.5 ± 26.5 (15)	79.0 ± 20.2 (8)	104.1 ± 26.1 (8)
120	73.9 ± 24.5 (17)	88.7 ± 26.9 (16)	85.8 ± 14.6 (9)	110.1 ± 26.1 (8)
150	69.7 ± 24.8 (18)	85.1 ± 22.4 (16)	89.5 ± 16.4 (8)	97.8 ± 20.7 (5)
180	71.6 ± 23.7 (17)	83.3 ± 27.6 (15)	83.4 ± 16.1 (8)	98.2 ± 27.8 (6)

*Plasma concentration of CGRP is expressed as mean ± standard deviation*.

### CGRP and VIP in Healthy Individuals

**Figure 3:**
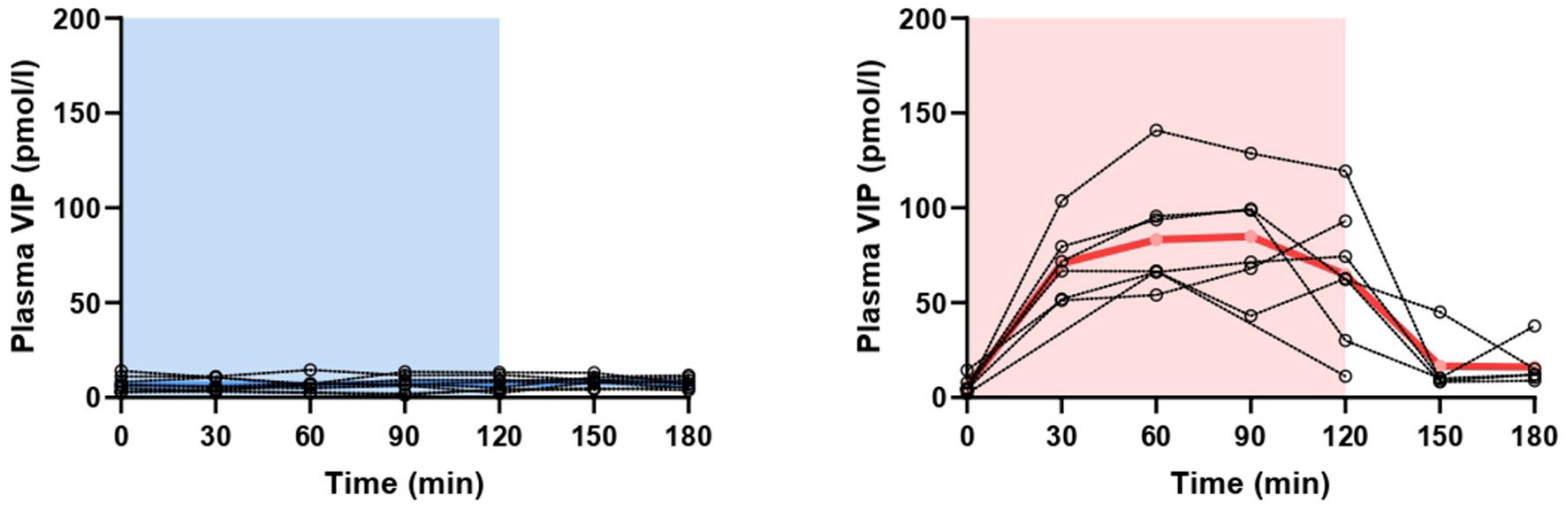
Changes in plasma concentration of vasoactive intestinal polypeptide (VIP) during and after 2-h infusion of placebo (*p* = 0.599) or VIP (*p* < 0.001) in healthy individuals. The light blue area represents placebo infusion, while the red area represents VIP infusion. Dotted lines represent individual values, while thick lines show mean concentrations.

On VIP days, plasma VIP was significantly higher compared to the baseline (*p* < 0.001), but not on placebo days (*p* = 0.599) (Figure 3). Peak concentration of plasma VIP was 84.9 ± 30.1 pmol/l, measured at T_90_ during VIP days. Results from the mixed effects analysis revealed no changes in plasma CGRP during VIP (*p* = 0.205) or placebo (*p* = 0.428) days. The mean plasma concentrations of CGRP in healthy individuals are reported in Table 1. A simple linear regression of plasma levels of CGRP during VIP and placebo days is displayed in Figure 2B.

### Baseline Concentration of Plasma CGRP

**Figure 4:**
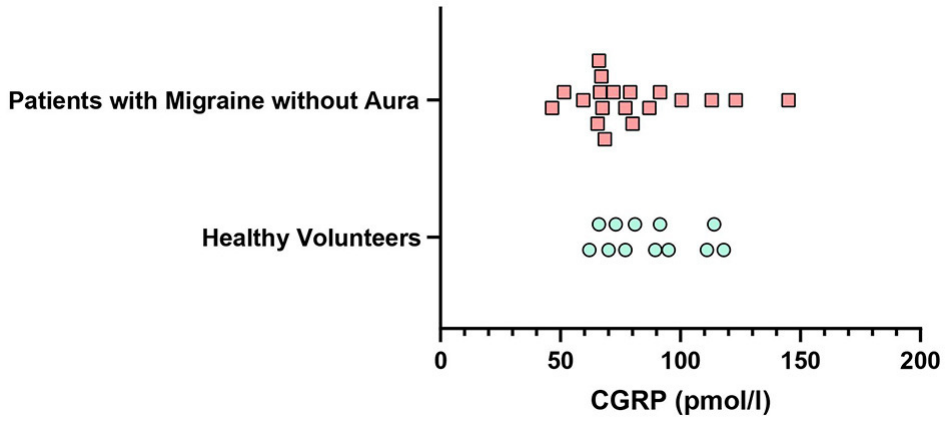
Mean plasma concentration of CGRP at the baseline in healthy individuals and patients with migraine (*p* = 0.232).

We found no difference in plasma levels of CGRP between healthy individuals (*n* = 12) (85.3 pmol/l, IQR 71.5–103.0) and patients with migraine (*n* = 19) (68.5 pmol/l, IQR 66.0–96.0) (*p* = 0.232). Individual baseline values are displayed in Figure 4.

## Discussion

The major finding of this study is that a 2-h infusion of VIP elevated plasma levels of CGRP in patients with migraine. However, plasma CGRP did not change during experimentally induced migraine attacks.

### VIP Effects on CGRP

This is the first human study investigating the effects of an infusion of VIP on plasma CGRP. The low volume of distribution of infused VIP (16) reflects a confined effect at the neurovascular interface, in disagreement with a direct stimulation of trigeminal ganglion neurons and satellite glial cells. Previously, one study reported elevated plasma levels of CGRP and VIP in two patients with migraine with aura during the headache phase and prominent symptoms of lacrimation and rhinorrhea (17). In our study, we found an increased plasma level of CGRP at T_30_ and T_60_ in patients with migraine, which occurred before the onset of migraine attacks. Recently, we proposed that VIP induces migraine through the vasoactive intestinal polypeptide receptor 1 (VPAC_1_) and the vasoactive intestinal polypeptide receptor 2 (VPAC_2_) (5). VPAC_1_ and VPAC_2_ are predominantly expressed in the smooth muscle cells of blood vessels (18–20). Theoretically, CGRP can be released from perivascular fibers after the binding of VIP to VPAC_1_ and VPAC_2_ that are expressed on vascular smooth muscle cells in their vicinity. However, only a limited number of studies have been conducted on the topic, with inconclusive results. In rats, an exogenous administration of VIP had no effect on CGRP release in dura mater, the trigeminal ganglion, and the trigeminal nucleus caudalis (21). In rodents, activation of vascular VPAC_1_ and VPAC_2_ resulted in the CGRP release from perivascular fibers (22–24). Most circulating CGRP comes from perivascular nerve terminals (25, 26) and its release might be favored by a prolonged vasodilation. In the present study, however, peripheral circulating levels of CGRP were unrelated to the observed migraine attacks. We cannot exclude the possibility of a local release (e.g., in dura mater or perivascular afferents) of CGRP in the cranium as it is not detectable by peripheral sampling. Despite this, we know that levels of circulating CGRP are not altered during attacks and did not differ between external jugular and peripheral venous blood when measured with the same assay (27). It would be interesting to investigate whether anti-CGRP therapies are able to prevent VIP-induced migraine attacks. It is also intriguing to note that VIP might directly activate the trigeminal system, leading to a secondary release of CGRP. However, VIP is very poorly distributed during an intravenous administration, and its inability to cross the blood-brain barrier points to a peripheral effect (16, 28).

### Interictal Levels of CGRP in Migraine

A previous study found that levels of plasma CGRP collected from the cubital vein were higher in patients with migraine caudal is without aura outside attacks (*n* = 14) than in healthy controls (*n* = 20) (29). Of interest, plasma CGRP concentrations were measured with the same radioimmunoassay technique reported in the present study. However, we did not replicate the same results. Our study was not powered to detect differences between patients and healthy controls. Moreover, the CGRP levels of our healthy individuals appeared unusually high, probably due to the smaller sample size (*n* = 12). Two other studies reported that plasma levels of CGRP did not differ between patients with episodic migraine and healthy individuals when blood was collected from the antecubital vein (30, 31). In a more recent study, interictal plasma CGRP did not differ between patients with episodic migraine and healthy individuals (32). The noticeable lack of reproducibility might stem from different study designs and assays. There are currently no defined gold standard assays for evaluation of plasma CGRP. Hence, the use of different assay techniques may have potentially biased findings of clinical importance. The consistent use of the same assay in adequately powered studies will provide more accurate information.

### Study Limitations

This study might have several limitations. First, our study is exploratory. We did not find any change in plasma CGRP during the induced migraine attacks. Several factors might explain these findings, including that no samples were collected at the onset of the attack. A retrospective assessment of the power of our analysis confirmed that with only six patients, the chance of detecting a real effect was low (α = 0.24). However, we believe that our analysis was a reasonable one for investigating the impact of a prolonged infusion of VIP on plasma CGRP, considering the presence of a placebo-controlled arm and the collection of blood samples from the same individual at different time points. While the current study design and the number of enrolled patients with migraine were sufficient to identify a direct effect of VIP on plasma CGRP, new studies will clarify whether this is of significance for migraine. We have also found no change of plasma CGRP in healthy individuals. Several factors might account for this result, including the low number of healthy individuals (*n* = 9). As *post-hoc* analysis, a simplified mixed effects model investigating only differences of plasma CGRP between T_0_, T_120_, and T_180_ in healthy individuals closely approaches statistical significance (T_0_ vs. T_120_, adjusted *p* value = 0.060). A larger number of participants might therefore show a significant increase of plasma CGRP in healthy individuals. Future studies with predefined outcomes and larger sample size will help to clarify the role of CGRP in VIP induced migraine.

## Conclusions

The present study showed elevated plasma levels of CGRP during a 2-h infusion of VIP in patients with migraine. However, the study revealed no relationship between plasma CGRP and VIP-induced migraine attacks. These findings generate further interest in clarifying the role of VIP and its receptors in migraine. Future studies will improve our understanding mechanisms underlying VIP-induced migraine attacks.

## Data Availability Statement

The original contributions presented in the study are included in the article/Supplementary Material, further inquiries can be directed to the corresponding author/s.

## Ethics Statement

The present studies (H-19075630 and H-18050862) were approved from the Regional Health Research Ethics Committee of the Capital Region. The patients/participants provided their written informed consent to participate in this study.

## Author Contributions

LP, MA-K, FA, and MA conceived and planned the experiments. LP, MA-K, RD, BC, and JH carried out the experiments. CL and JS contributed to the design of the research and interpretation of the results. LP analyzed the data and took the lead in writing the manuscript with input from all authors. All authors provided critical feedback and helped shape the research, analysis, and manuscript. All authors contributed to the article and approved the submitted version.

## Funding

MA was supported by the Lundbeck Foundation professor grant (R310-2018-3711). This study received funding from Novartis Pharma AG.

## Conflict of Interest

MA reported receiving personal fees from AbbVie, Allergan, Amgen, Eli Lilly, Lundbeck, Novartis, and Teva Pharmaceuticals during the conduct of the study. MA reported serving as Associate Editor of Cephalalgia, The Journal of Headache and Pain and Brain. FA is principal investigator for a phase IV trial for Teva. FA has received personal fees for lecturing and/or participating in advisory boards for Teva, Novartis, Eli Lilly and Lundbeck. CL reported being full employee at Roche Holding AG and shareholder of Novartis International AG. JS reported being full-time employee and shareholder of Novartis International AG. MA-K reported being an invited speaker for Novartis and receiving fees from ElectroCore. JH reported receiving fees from the Danish Biotechnology Center for Cellular Communication. CL and JS were employed by Novartis Pharma AG. This study received funding from Novartis Pharma AG. The funder had the following involvement with the study: support for study design and critical revision of the manuscript.

## Publisher's Note

All claims expressed in this article are solely those of the authors and do not necessarily represent those of their affiliated organizations, or those of the publisher, the editors and the reviewers. Any product that may be evaluated in this article, or claim that may be made by its manufacturer, is not guaranteed or endorsed by the publisher.
