# Supplementary figures and images for: Plasma Levels of CGRP During a 2-h Infusion of VIP in Healthy Volunteers and Patients With Migraine: An Exploratory Study
Source: Front Neurol. 2022 Apr 1;13:871176. doi: 10.3389/fneur.2022.871176 (PMC9011105; doi:10.3389/fneur.2022.871176)

## Patients with migraine (NCT04260035)

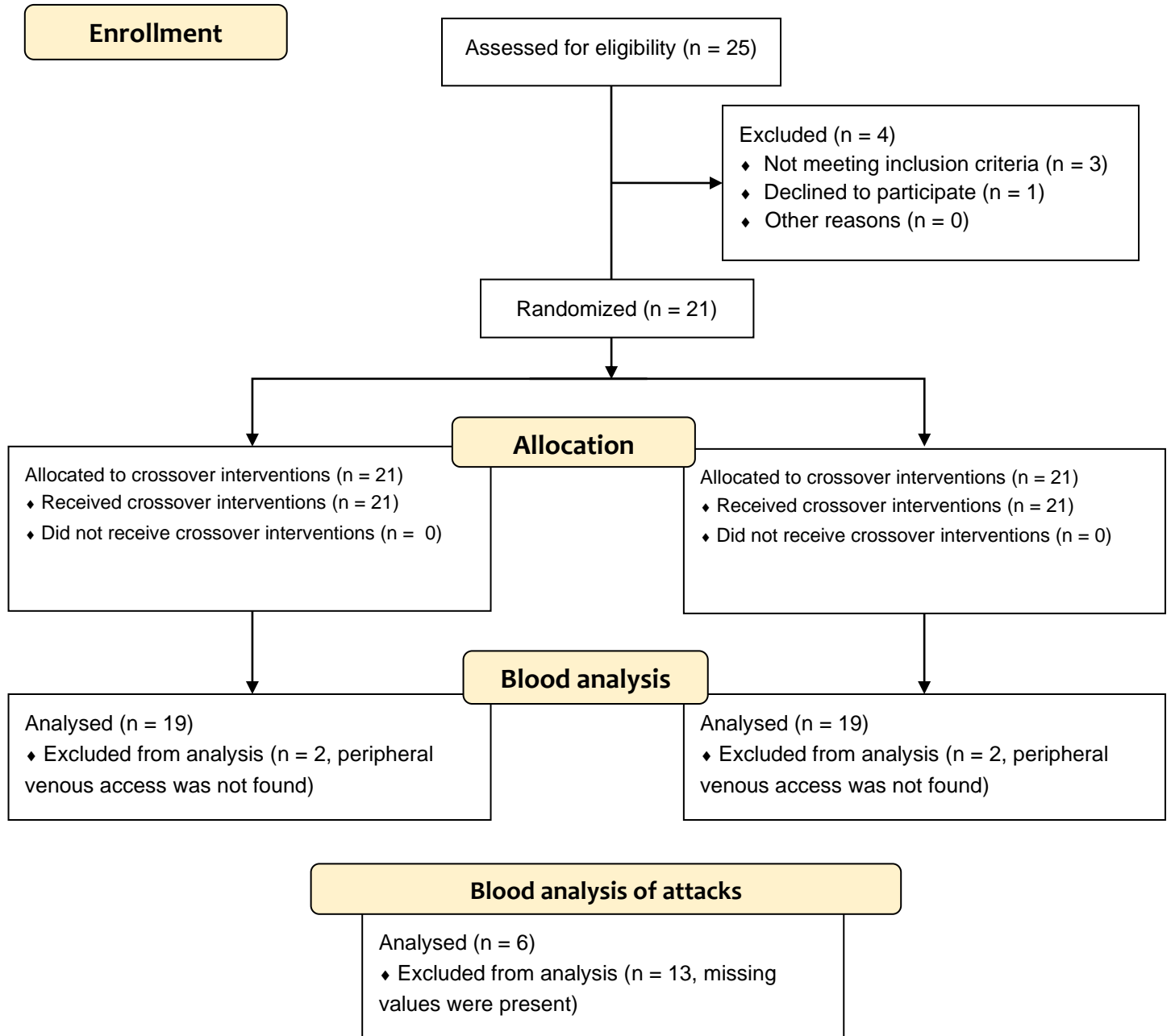

Supplement: Supplementary file 2 [file Data_Sheet_2.PDF]

## Healthy Volunteers (NCT03989817)

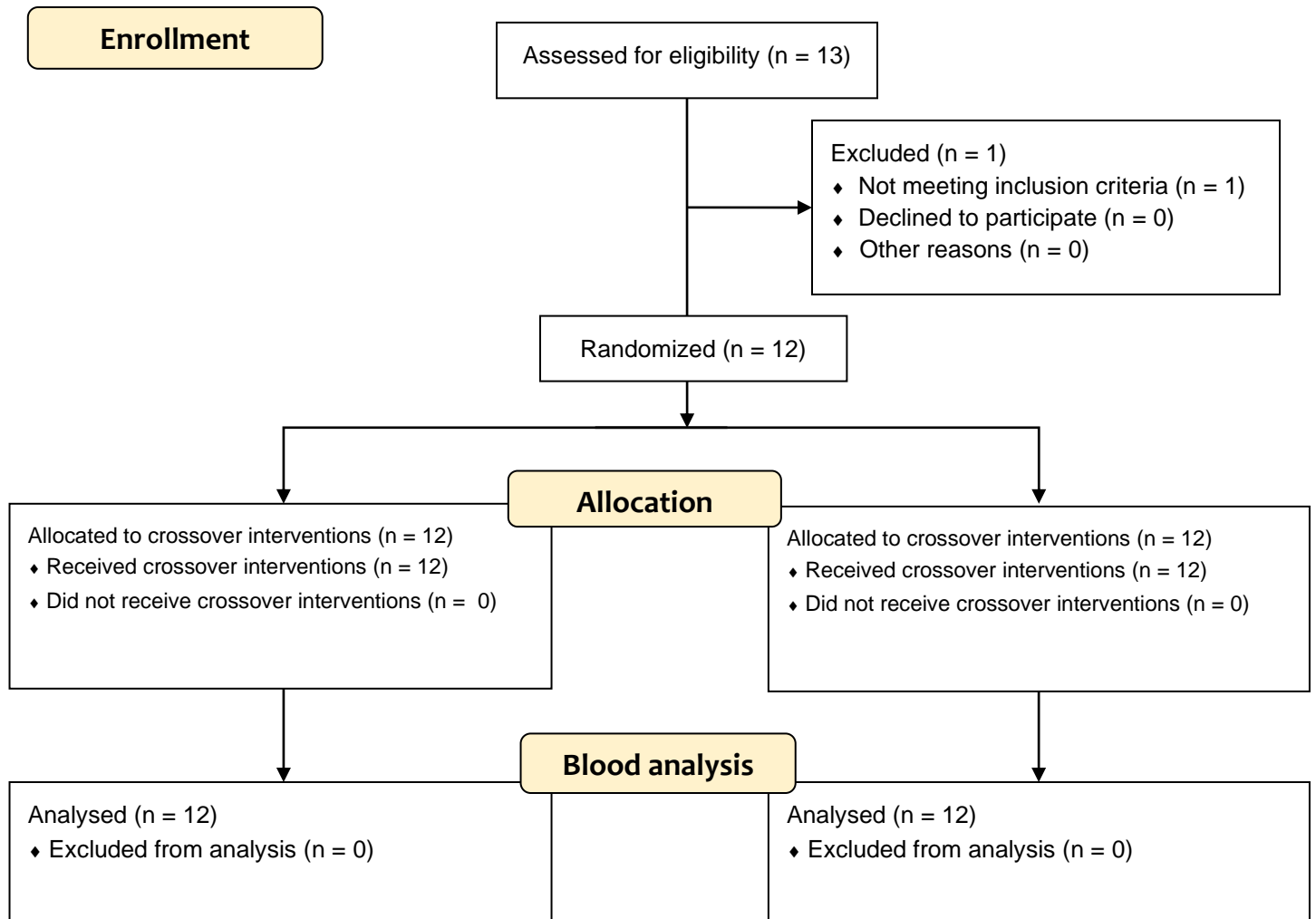

Supplement: Supplementary file 3 [file Data_Sheet_3.PDF]
